# Performance improvement of a tunnel junction memristor with amorphous insulator film

**DOI:** 10.1186/s11671-023-03800-0

**Published:** 2023-02-21

**Authors:** Fenning Liu, Yue Peng, Yan Liu, Wenwu Xiao, Yue Hao, Genquan Han

**Affiliations:** 1 State Key Discipline Laboratory of Wide Band Gap Semiconductor Technology, School of Microelectronics, Xidian University, Xi’an, 710071 People’s Republic of China; 2 Emerging Device and Chip Laboratory, Hangzhou Institute of Technology, Xidian University, Hangzhou, 311200 People’s Republic of China; 3 Xi’an UniIC Semiconductors, Xi’an, 710075 China

**Keywords:** Tunneling junction, TJM, Oxygen vacancy, Memristor, Tunneling electroresistance

## Abstract

This study theoretically demonstrated the oxygen vacancy (*V*_O_^2+^)-based modulation of a tunneling junction memristor (TJM) with a high and tunable tunneling electroresistance (TER) ratio. The tunneling barrier height and width are modulated by the *V*_O_^2+^-related dipoles, and the ON and OFF-state of the device are achieved by the accumulation of *V*_O_^2+^ and negative charges near the semiconductor electrode, respectively. Furthemore, the TER ratio of TJMs can be tuned by varying the density of the ion dipoles (*N*_dipole_), thicknesses of ferroelectric-like film (*T*_FE_) and SiO_2_ (*T*_ox_), doping concentration (*N*_d_) of the semiconductor electrode, and the workfunction of the top electrode (TE). An optimized TER ratio can be achieved with high oxygen vacancy density, relatively thick *T*_FE_, thin *T*_ox_, small *N*_d_, and moderate TE workfunction.

## Intoduction

Novel two-terminal memory types mainly include Phase Change Random Access Memory (PCRAM), Magnetic Random Access Memory (MRAM), and Resistive Random Access Memory (RRAM). Specitically, the resistance switching of PCRAM is realized by altering the phase change materials between crystalline and amorphous states. The switching ratio is up to 10^5^, but the crystallization temperature of the material is high, and large power consumption is required due to the current-drive switching [1]. The resistance of MRAM is realized by modulating the spin direction of the free layer, showing excellent endurance characteristics. Its switching ratio is, however, too small (usually only several times) [2]. The RRAM is realized by forming and fracturing a conductive filament in peroxides, its processes are unstable and result in large variations between the RRAM devices [3]. In addition, studies identified another two-terminal memory—the charge trapping memory, whose switching between high and low capacitance states is based on charge trapping [4, 5].

Besides, the two-terminal tunneling junction memristor (TJM) is another kind of promising device candidate in next-generation memories, owing to its attractive advantages, such as high density data storage, fast access speed, and low power consumption [6, 7]. Among various nonvolatile TJMs, the ferroelectric tunneling junction (FTJ) exploiting the polarization-dependent tunneling electroresistance (TER) effect has specifically attracted much attention [8, 9]. An FTJ consists of a thin ferroelectric barrier sandwiched between two electrodes with different work functions and screening lengths,its barrier profile can be modulated by polarization switching. Enormous research efforts have been devoted to improving the TER ratio in TJMs based on the HfO_2_-based polycrystalline ferroelectrics [10] and the traditional perovskite materials [11–14]. However, the the TER ratio of the current HfO_2_-based TJMs is not more than 100 [15]. One practical feasible way is to decrease the OFF-state tunneling current by employing an amorphous tunneling barrier [16]. Utilizing modulated tunneling barrier width technique, the BTO TJM has exhibited a TER ratio above 10^6^ [17], but which suffers from the incompatibilities during the Si CMOS process.

Some amorphous films, including Al_2_O_3_, ZrO_2_, HfO_2_, and La_2_O_3_ were also reported to have ferroelectric-like properties providing the non-volatile electrical performance in metal/insulator/metal (semiconductor) capacitors and field-effect transistors [18–22]. The underlying mechanism is supposed to be the movement of oxygen vacancy (*V*_O_^2+^) dipoles or mobile ions under applied voltages, which is different from the ferroelectric properties of HfO_*x*_-based films induced by the orthorhombic phase. The previous experiments have shown that amorphous film based mobile-ionic TJM achieved a decent TER ratio enabled by the modulated tunneling barrier width by mobile ions [16].

Therefore, based on the above background, this work theoretically characterized the TJMs modulated by *V*_O_^2+^-related dipoles under the external electric fieldand via TCAD simulation. Physical mechanism underlying performance improvement in the devices and its dependence on dipole density (*N*_dipole_), thicknesses of ferroelectric-like film (*T*_FE_) and SiO_2_ (*T*_ox_), doping concentration (*N*_d_) of semiconductor electrode, and workfunction of top electrode (TE) are investigated.

## Methods

**Fig. 1:**
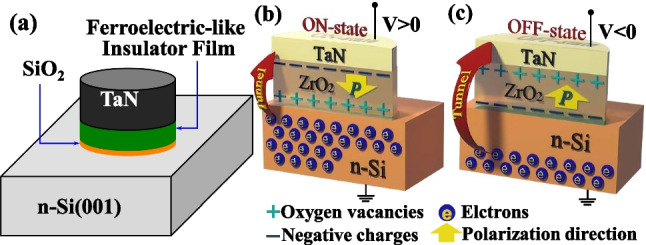
**a** Three-dimensional schematic of the TJM with metal/insulator/SiO_2_/Si structure. Schematics of the mechanism for the tunable tunneling barrier width at the **b** ON- and **c** OFF-states by the switchable *V*_O_^2+^-related dipoles, respectively

**Fig. 2:**
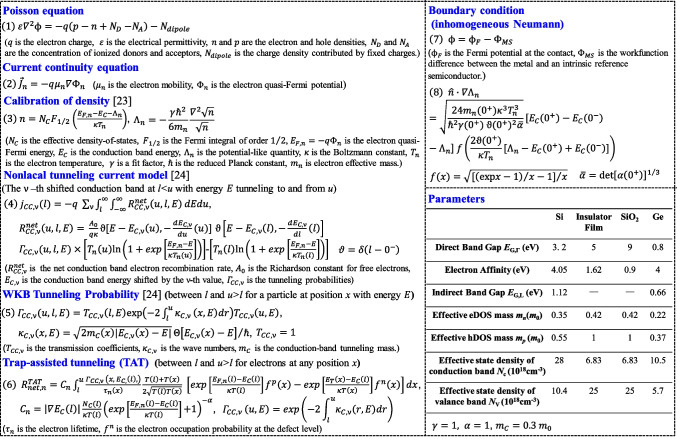
Schematic showing the simulation framework, including the modeling formulas, parameters, and boundary conditions for the simulation of TJMs

**Fig. 3:**
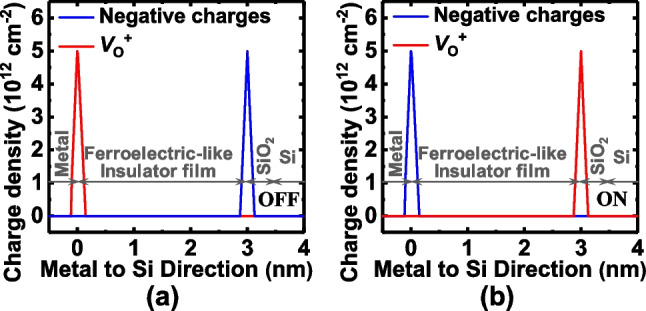
The distribution of *V*_O_^2+^ and negative charges at **a** ON- and **b** OFF-states

Figure 1a shows the three-dimensional schematic of the proposed TJM consisting of a top metal electrode, ferroelectric-like insulator film, and bottom Si electrode. A SiO_2_ interfacial layer forms between oxide insulator film and Si. The thicknesses of the insulator film and SiO_2_ interfacial layer are 3 and 0.5 nm, respectively. Figure 1b and c show the underlying mechanism for the TER effect in TJM. The *V*_O_^2+^ and negative charges forming the electric dipoles, can be modulated with the applied voltage providing a tunable tunneling barrier width in the device [16]. The 2D numerical simulation is carried out by sentaurus TCAD tool, realizing a dynamic nonlocal tunneling algorithm which is used to describe the tunneling at the interfaces and junctions. The complete coupling method of the quantum modified current continuity equation and Poisson equation is adopted for calculating the energy band alignment. The quantum model (density gradient quantum correction model) which considers the quantization of carriers near the tunneling junction is mainly used to accurately calculate the carrier density and distribution. The method for introducing quantization effects to the classical model is to introduce a potential-like quantity Λ_n_, where Λ_n_ is calculated through the density gradient quantum correction model by Eq. (3) in Fig. 2. Once obtained the band alignment, the tunneling current is calculated using the nonlocal tunneling model based on the Wentzel-Kramers-Brillouin (WKB) approximation, and the nonlocal path trap-assisted tunneling (TAT) is also considered. Figure 2 shows the simulation framework, detailed formulations, parameters, and boundary conditions. Some other models, such as the high field velocity saturation model, doping-dependent mobility model, and Shockley Read Hall generation recombination model. The doping-dependent mobility model used in the simulation was proposed in [25]. The high-field saturation model was from a Canali model [26]. In addition, the distributions of *V*_O_^2+^ and negative charges at ON- and OFF- states are assumed to be a Gaussian type, as shown in Fig. 3a and b, respectively. Since studies previously reported that while that *V*_O_^2+^ was formed during the deposition of oxide films, it was also generated due to the scavenging effect with the formation of the TaON interfacial layer [27, 28]. The negative charges can be related to the metal ion vacancy and, eg. Zr vacancy for ZrO_2_ film, and Al vacancy for Al_2_O_3_ film [29, 30], and the negative charges are mobile [31–33]. The underlying mechanism for the *P–V* loops is the switching of dipoles formed by the *V*_O_^2+^ and negative charges, which is a long range *P* switching during the electric field cycling [34].

## Results and discussion

**Fig. 4:**
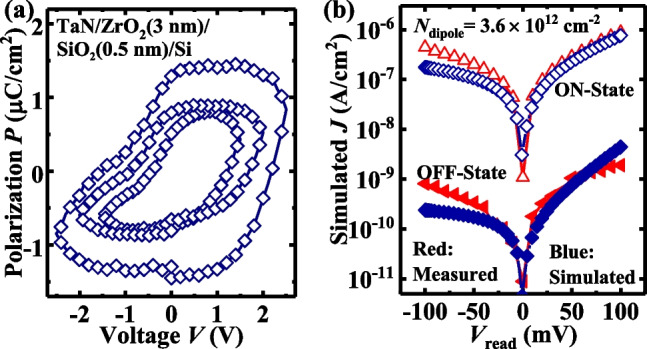
**a** Measured *P–V* curves for TaN/ZrO_2_/SiO_2_/Si capacitor. **b** *J–V*_read_ curves for the measured and simulated TJM at ON-state and OFF-state

Figure 4a shows the measured *P–V* curves for the TaN/ZrO_2_/SiO_2_/Si capacitor, the *P*_r_ values are 0.57, 0.82 and 1.29 μC/cm^2^ for the voltage of 1.5, 2 and 2.5 V, respectively. Figure 4b shows the measured and simulated *J–V*_read_ curves of TJM, in which the measured curves at ON-state and OFF-state are read after the pulses of + 1.5 V/1 μs and − 1.5 V/1 μs, respectively. The simulated results were based on a *N*_diople_ of 3.6 × 10^12^ cm^−2^, which is determined by the polarization 0.57 μC/cm^2^ under 1.5 V. Obviously, the experimental results were consistent with the simulated ones.

### The resistive switch enabled by modulating dipoles in the insulator

**Fig. 5:**
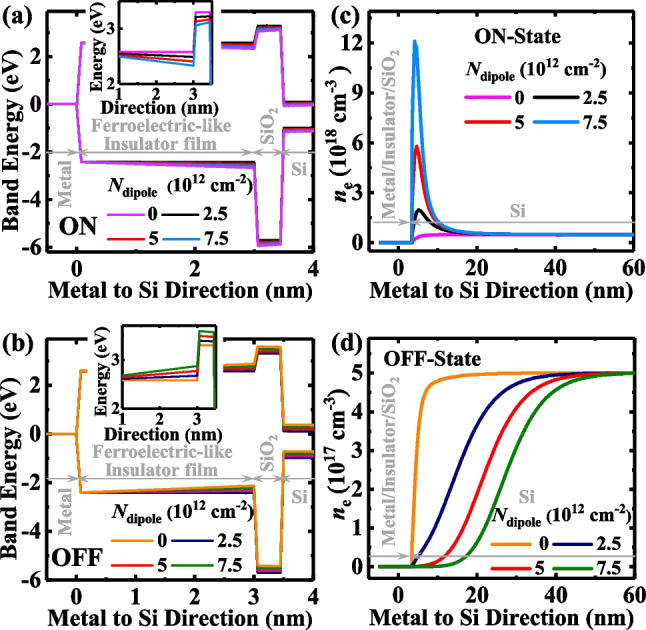
Energy band diagrams for the TJMs at **a** ON-state and **b** OFF-state with *N*_dipole_ ranging from 0 to 7.5 × 10^12^ cm^−2^. The Insets show the zoomed-in results at the insulator/SiO_2_ interface. The electron distribution in Si at **c** ON-state and **d** OFF-state with various *N*_dipole_

To get a deep insight into how the *V*_O_^2+^-related dipoles modulate the tunneling barrier height and width of the amorphous TJMs, we plot energy band diagrams and electron concentration distributions at a zero-external voltage of TJM at ON-state and OFF-state. As shown in Fig. 1b and c, the ON-state and OFF-state of the device are enabled by the accumulation of *V*_O_^2+^ and negative charges, respectively, near the amorphous oxide/SiO_2_ interface. Figure 5a and b show the energy band diagrams at the ON-state and OFF-states, respectively, and the insets show the zoomed-in view of the conduction band at the insulator/SiO_2_ interface. Notably, the electrostatic potential profile for tunneling of amorphous layer induced by the accumulation of *V*_O_^2+^ near the amorphous oxide/SiO_2_ interface is lower than that induced by the negative charges. Figure 5c and d show the electron concentration distributions at the ON- and OFF-states, respectively. We observed that the *V*_O_^2+^-related dipoles affected the distribution of carriers in the semiconductor electrode, directly determining the lateral tunneling path of TJM. Furthermore, the accumulation of positive/negative charges near the amorphous oxide/SiO_2_ interfaces tugs/pushes the electrons toward/away from the TE decreasing/increasing the tunneling path. Moreover, the concentration of the *V*_O_^2+^-related dipoles increased, the effect of lateral tunneling path modulation became better.

**Fig. 6:**
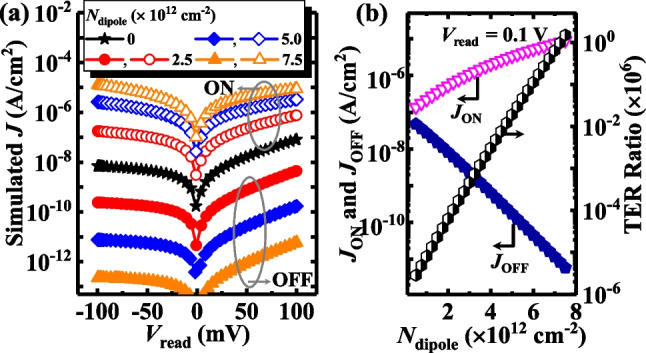
**a** *J*–*V*_read_ curves of TJM with *N*_dipole_ at ON-state and OFF-state. **b** *J*_ON_, *J*_OFF_ and TER Ratio of TJM versus *N*_dipole_

Figure 6a plots the simulated current density (*J*) versus the read voltage (*V*_read_) for the TJMs with the different *N*_diople_, demonstrating the *J* is effectively determined by the direction and concentration of the dipoles. Figure 6b shows the extracted ON-state current density (*J*_ON_), OFF-state current density(*J*_OFF_), and the TER ratio versus *N*_dipole_ at a *V*_read_ of 0.1 V, the *N*_dipole_ varies from 0 to 7.5 × 10^12^ cm^−2^, with a step of 0.25 × 10^12^ cm^−2^. We also observed the *J*_ON_ increases and *J*_OFF_ decreases as *N*_dipole_ increased,ncluding an exponential increase in the TER ratio of the TJMs with *N*_dipole_. The TER ratio of the device with a *N*_dipole_ of 7.5 × 10^12^ cm^−2^ is 10^6^ times higher compared to the TJM with a *N*_dipole_ of 0.5 × 10^12^ cm^−2^. It is also seen that *J*_OFF_ descends at a faster rate compared to the variation of *J*_ON_, showing that the dipole has a greater influence on the OFF-state, corresponding with the results in the above-mentioned energy band diagram results.

Studies have previously reported that the density of *V*_O_^2+^-related dipoles can be optimized by changing the deposition condition of the insulator film [35–37]. Experiments have demonstrated that the post-deposition annealing of the device structure can induce a scavenging effect, giving rise to the formation of oxygen vacancies, *i.e.* positive ions, in the insulator [38]. It has also been proposed that although TJM is more sensitive to temperature at more oxygen vacancies, allowing for the temperature-dependent mobility of oxygen vacancies and phonon-assisted detrapping process [39, 40], lower temperatures can inhibit the accumulation of oxygen vacancies at the FE/SiO_2_ interfaces.

### Performance dependence on ***T***_*FE*_ and ***T***_***ox***_

**Fig. 7:**
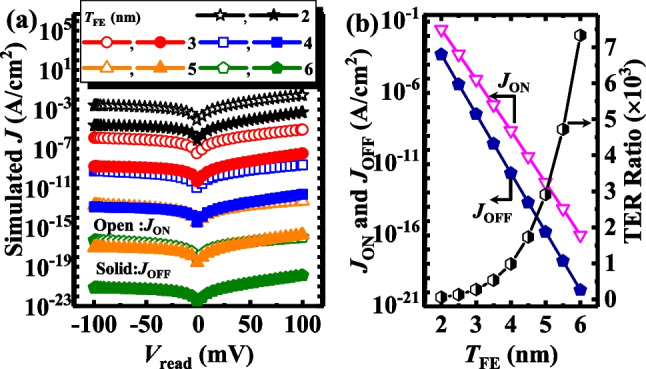
**a** *J*–*V*_read_ curves of TJM with different *T*_FE_ at ON-state and OFF-state. **b** *J*_ON_,* J*_OFF_ and TER ratio of TJM versus *T*_FE_

**Fig. 8:**
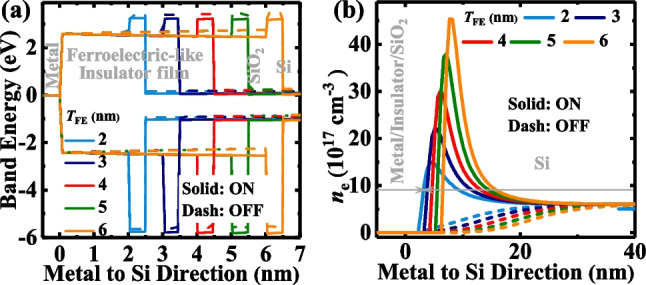
**a** Energy band diagrams for the TJMs at ON-state and OFF-state with different *T*_FE_. **b** The electron distributions in Si at ON- and OFF-states with various *T*_FE_

Since the resistive mechanism could be realized by switching the distribution of *V*_O_^2+^-related dipoles, we also investigated the effects of the* T*_*FE*_* and T*_*ox*_ on the performance of the TJM devices to confirm this hypothesis. Figure 7a shows the *J*_ON_ and *J*_OFF_ for TJM with different *T*_*FE*_ values of 2–6 nm. The open symbols represent *J*_ON_ and the solid ones represent* J*_OFF_. Here, *N*_d_ and *N*_dipole_ values of the devices are fixed at 5 × 10^17^ cm^−3^ and 2.5 × 10^12^ cm^−2^, respectively, and the TE workfunction is 4.3 eV. Figure 7b extracts the *J*_ON_ and *J*_OFF_ and TER ratio versus *T*_FE_.Obviously, while *J* was degraded with an increase in *T*_FE_, the TER ratio increased as *T*_FE_ increased, proposed to be caused by the faster decrease of *J*_OFF_ due to the more enhanced modulation effect of the tunneling barrier width at the OFF-state. To get a deep insight, the energy band diagrams and electron concentration distributions at a zero-external voltage of TJM at ON-state and OFF-state are plotted in Fig. 8a and b. The barrier height decreases and increases at ON-state and OFF-state with the increase of *T*_FE_, respectively, which is the reason for the increased TER ratio. In Fig. 8b, the number of electrons accumulated and exhausted at ON- and OFF-states increases as the increase of *T*_FE_, making the tunneling barrier width at the two states wider. Moreover, compared with the ON-state, the barrier width at OFF-state changed more with an increase in *T*_FE_, explaining the increased TER ratio. Therefore, we establish that a thicker *T*_FE_ improved the TER ratio by promoting the modulation effect of tunneling barrier, which is due to the higher voltage distribution on the ferroelectric layer.

**Fig. 9:**
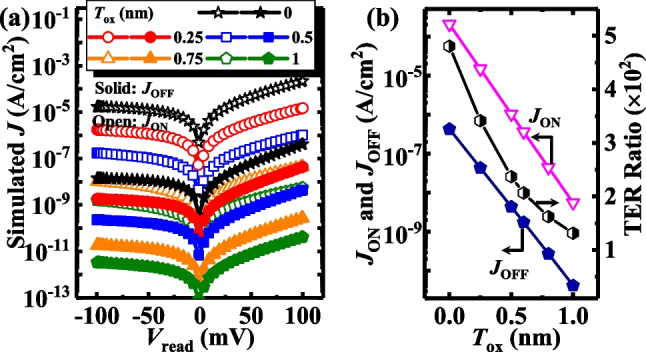
**a** *J–V*_read_ curves of TJM with different *T*_ox_ at ON-state and OFF-state. **b** *J*_ON_,* J*_OFF_ and TER ratio of TJM versus *T*_ox_

**Fig. 10:**
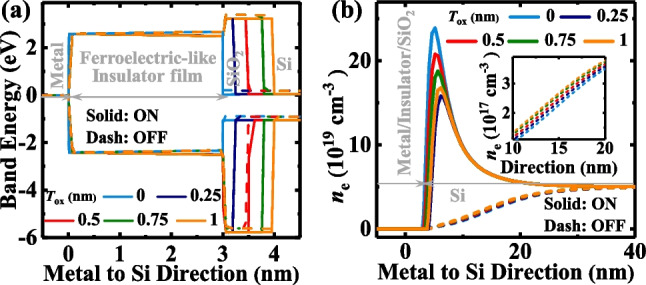
**a** Energy band diagrams for the TJMs at ON-state and OFF-state with different *T*_ox_. **b** The electron distributions in Si at ON- and OFF-states with various *T*_ox_. The inset shows the zoomed-in results in Si

Figure 9 shows the effect of *T*_ox_ on the performance of the TJM. Figure 9a shows the *J*_ON_ and *J*_OFF_ of the TJM at different *T*_ox_ of 0, 0.25, 0.5, 0.75, and 1 nm. Similarly, *N*_d_ and *N*_dipole_ values of the devices are fixed at 5 × 10^17^ cm^−3^ and 2.5 × 10^12^ cm^−2^, respectively, and the TE workfunction is 4.3 eV. Obviously, at a fixed *V*_read_ of 0.1 V, both *J*_ON_ and *J*_OFF_ decreased with an increasing *T*_ox_, which is also shown in Fig. 9b. This was caused by the slower decrease of *J*_OFF_ due to the more reduced modulation effect of the tunneling barrier width at the OFF-state. The energy band diagrams and electron concentration distributions at a zero-external voltage of TJM at ON-state and OFF-state are plotted in Fig. 10a and b to get a deep insight. The barrier heights are almost constant and the tunneling widths in the SiO_2_ increase with an increase in *T*_ox_ at both the ON- and OFF-states, which is the reason for the decreased *J*. Furthermore, the tunneling widths in Si are constant at the ON-state, but narrow with an increase in *T*_ox_ at the OFF-state, indicating that the modulation effect on semiconductor depletion is weakened as the *T*_ox_ increases, blocking the *J*_OFF_ decrease, and thus decreasing the TER ratio.

### Performance dependence on ***N***_d_

**Fig. 11:**
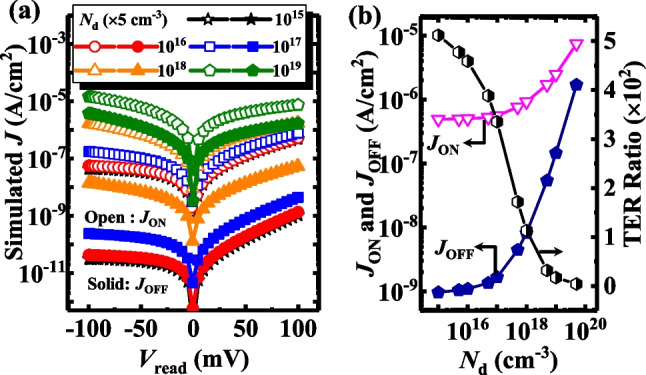
**a** *J*–*V*_read_ curves of TJM with different *N*_d_ at ON-state and OFF-state. **b** *J*_ON_, *J*_OFF,_ and TER Ratio of TJM versus *N*_d_

Figure 11 shows the effect of *T*_FE_ on the performance of the TJM. Figure 11a shows the *J*_ON_ and *J*_OFF_ of the TJM with different *N*_d_ values of 5 × 10^15^, 10^16^, 10^17^, 10^18^, and 10^19^ cm^−3^, The open symbols represent *J*_ON_ and the solid ones represent *J*_OFF_. Here, the *N*_dipole_ of the devices is fixed at 2.5 × 10^12^ cm^−2^ and the TE workfunction is 4.3 eV. Obviously, at a fixed *V*_read_, both *J*_ON_ and *J*_OFF_ increase with increasing *N*_d_, as shown in Fig. 11b. Similarly, we extract the TER ratio of the devices at a *V*_read_ of 0.1 V, the TER ratio decreases with an increase in *N*_d_. As the value of *N*_d_ increases to ~ 10^20^ cm^−3^, the TER ratio is less than 10, proposed to be due to the reduced modulation effect of tunneling barrier height and width with the thin depletion layer and the short screen length of the heavily doped Si.

**Fig. 12:**
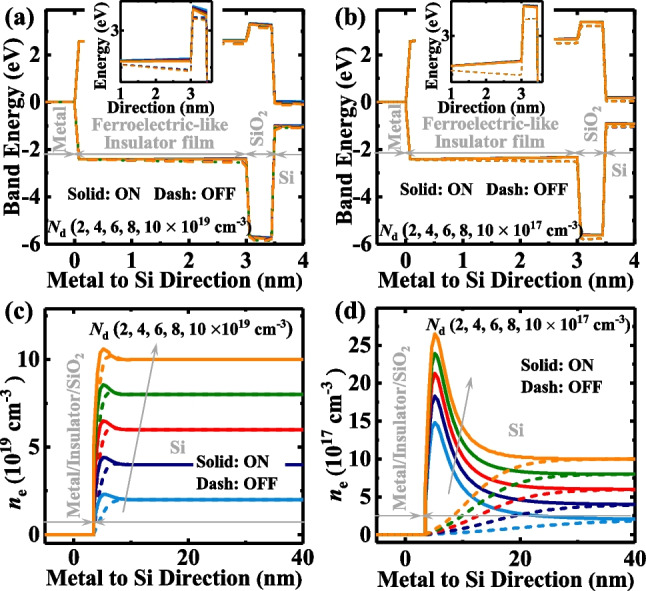
Energy Band diagrams for the TJMs with different *N*_d_ **a** (*N*_d_ = 2, 4, 6, 8, and 10 × 10^19^ cm^−3^) and **b** (*N*_d_ = 2, 4, 6, 8, and 10 × 10^17^ cm^−3^). The Insets show the zoomed-in results at the insulator/SiO_2_ interface. **c** and **d** The electron distributions in Si with various *N*_d_

Figure 12a and b show the energy band diagrams of the TJMs for different *N*_d_ for heavily and lightly doped substrates, respectively. Figure 12c and d show the *n*_e_ profiles of the TJMs with various *N*_d_ for heavily and lightly doped substrates, respectively. The modulation of the tunneling barrier width is pronouncedly reduced with increased *N*_d_ in the semiconductor electrode for both the ON- and OFF-states. The increased *N*_d_ dramatically heightened the OFF-state’s current, degrading the standby power performance of the device. Furthermore, the reduced TER ratio due to the heavy doping will dangerously increase the reading error of the circuit. However, we can get the bonus of using the heavily doped Si—reducing the required thickness of the electrode to increase the vertical integration density of the circuit.

### Impact of TE workfunction on device performance

**Fig. 13:**
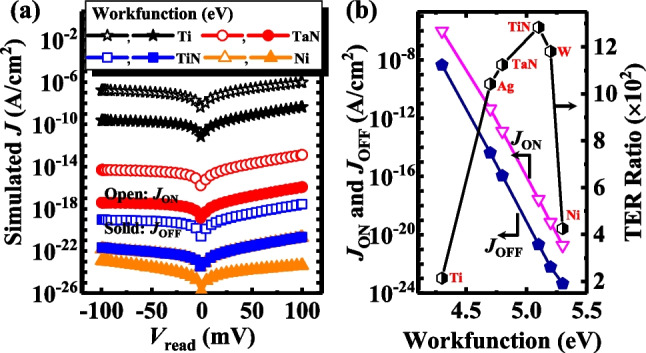
**a** *J*–*V*_read_ curves of TJMs with different TE workfunctions at ON-state and OFF-state. **b** *J*_ON_, *J*_OFF_, and TER Ratio versus TE workfunction for the devices. The workfunction values of the metals are taken from Refs. [41, 42]

Finally, we also investigated the dependence of the amorphous TJM device performance on the TE workfunction. Here, *N*_dipole_ is fixed at 2.5 × 10^12^ cm^−2^ and *N*_d_ is 5 × 10^17^ cm^−3^. Figure 13a shows the *J*_ON_ and *J*_OFF_ versus read voltage curves for the TJMs with different TE workfunctions corresponding to Ti (4.3 eV), TaN (4.8 eV), TiN (5.1 eV), and Ni (5.3 eV). At a fixed *V*_read_, both *J*_ON_ and *J*_OFF_ decrease as the TE workfunction increases, which is mainly due to the raised tunneling barrier height and widened tunneling barrier, as shown in Fig. 9.

Simiarly, we extract the TER ratios of the devices at *V*_read_ = 0.1 V, the TER ratio first increases and then decreases with an increase in the TE workfunction. If the TE workfunction is too small/large, large quantities of negative/positive charges will accumulate at the interface of SiO_2_/Si, leading to a deep accumulation/depletion state of the semiconductor. However, it is difficult for *V*_O_^2+^-related dipoles with limited densities to modulate the deep accumulation/depletion of the semiconductor, as shown in the electrostatic potential profiles for Ti and Ni in Fig. 13a. Therefore, we propose the requirenment of a moderate TE workfunction to achieve a high TER ratio. Investigations prove TiN to be the best choice for this model and this method is the easiest among the three optimizations in the experiment.

**Fig. 14:**
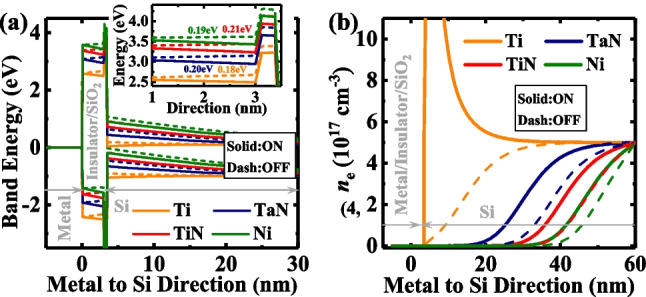
**a** Energy band diagrams for the TJMs at ON-state and OFF-state with different TE workfunctions. The inset shows the zoomed-in results at the insulator/SiO_2_ interface. **b** The electron distributions in Si at ON-state and OFF-state with various TE workfunctions

Furthermore, as shown in Fig. 14a and b, the influence of the dipoles on tunneling barrier height increase firstly and then decreases as the workfunction increases, and that on tunneling barrier width weakens with the increase of workfunction. Investigations also revealed that the varying trend of the TER ratio with work function is consistent with that of barrier height, indicating that the effect of TE workfunction on TJM is mainly affected by the tunneling barrier height and modulated by *V*_O_^2+^-related dipoles.

## Conclusions

The TJMs modulated by the *V*_O_^2+^-related dipoles under the external electric field are theoretically characterized by the numerical TCAD simulation. The physical mechanism underlying the performance improvement in the devices and its dependence on *N*_dipole_, *N*_d_ of the semiconductor electrode, and TE workfunction are investigated by comparing the *J* versus *V*_read_ curves, energy band diagrams, and *n*_e_ profile. It is demonstrated that an optimized TER ratio can be achieved with small *N*_d_, thick *T*_FE_, thin *T*_ox_, high oxygen vacancies density, and moderate TE workfunction.

## Data Availability

The datasets supporting the conclusions of this article are included in the article.

## Abbreviations

*V*_O_^2+^: Oxygen vacancy
TJM: Tunneling junction memristor
TER: Tunneling electroresistance
*N*_dipole_: Ion dipoles
*N*_d_: Doping concentration
TE: Top electrode
FTJ: Ferroelectric tunneling junction
Al_2_O_3_: Aluminum oxide
ZrO_2_: Zirconia oxide
La_2_O_3_: Lanthanum oxide
HfOx: Hafnium oxide
3D: Three-dimensional
SiO_2_: Silicon oxide
Si: Silicon
2D: Two-dimensional
TCAD: Technology computer aided design
Λ_n_: Potential-like quantity
WKB: Wentzel–Kramers–Brillouin
TAT: Trap-assisted tunneling
TaON: Tantalum oxynitride
Zr: Zirconia
Al: Aluminum
*P*: Polarization
*V*: Voltage
*J*: Current density
*V*_read_: Read voltage
*J*_ON_: ON-state current density
*J*_OFF_: OFF-state current density
FE: Ferroelectric-like film
*n*_e_: Electron concentration
Ti: Titanium
TaN: Tantalum nitride
TiN: Titanium nitride
Ni: Nickel
